# Impact of climate change on sensitive marine and extreme terrestrial ecosystems: Recent progresses and future challenges: *This article belongs to* Ambio*’s 50th Anniversary Collection. Theme: Climate change impact*

**DOI:** 10.1007/s13280-020-01446-1

**Published:** 2021-03-01

**Authors:** Deliang Chen

**Affiliations:** Regional Climate Group, Department of Earth Sciences, University of Gothenburg, Gothenburg, Sweden

**Keywords:** Arctic, Climate change, Coral bleaching, Science-based policy, Snow, glacier and permafrost, The European Alps

## Abstract

Climate change is the greatest global threat to ecosystems on the Earth. Previous studies assessed the impacts of climate change on sensitive tropical coral reefs, extreme environments in European Alps and the Arctic with a focus on snow and permafrost. This article reflects on the past developments and future challenges for scientific research and policy response relating to these topics from a peer’s perspective. This leads to the identification of several warning signs for potentially dangerous developments in these sensitive system and extreme environments as well as opportunities for research and policy in the future. While urgent actions are required to limit global warming, science-based policy can provide needed guidance.

## Sensitive marine and extreme terrestrial ecosystems threatened by climate changes

Scientific theory and observational evidence indicate that the Earth system has been warming, and that these changes are primarily due to greenhouse gases derived from human activities. Climate change poses a serious threat to ecosystems on the Earth, both on the land and in the seas. The impact of climate change on sensitive systems or systems in extreme climatic conditions (polar and high mountain regions) are expected to be felt earlier and stronger.

Coral reef ecosystems are among the most sensitive marine systems to climate change, and Goreau and Hayes (1994) found that coral bleaching was closely linked to high sea surface temperature which was getting higher under global warming. The European Alps and the Arctic belong to the most extreme environments that have undergone significant changes. Haeberli and Beniston (1998) pointed out that the Alps experienced more warming than the global mean, which caused melting of the glaciers and permafrost, while Callaghan et al. (2004, 2011) established that Arctic climate in terms of temperature, precipitation rate and snow structure, as well as ultraviolet radiation (UV-B) has experienced changes that will likely change the distributions of the plant and animal species.

## What happened after the publication of the articles?

The last decade has witnessed a number of progresses related to the issues identified by the four articles. First, much more monitoring data, especially those from satellite and models, have been collected and analyzed, leading to more accurate determination of relevant changes in the environment including climate. The lack of observations and the need for long-term monitoring were pointed out by these early works, which has pushed the research front forward. Second, models used to describe relevant processes in the Earth System and to predict climate change have been significantly improved. Third, the combination of the data and model developments, together with in-depth disciplinary studies and international and interdisciplinary cooperation have led to significantly enhanced understanding of how the climate has changed in the past and how it will change in the future, as well as how these changes could interact with non-climate factors to shape the ecosystem and the environments.

According to a recent report from the National Academies of Sciences, Engineering and Medicine (2019), coral reef coverage around the world has declined by 30–50% since the 1980s. Nearly 75% of the world’s reefs face threats from environmental changes, and increasingly, a warming climate. In addition to the warming, we understand now that factors such as high solar irradiance, ocean acidification, habitat destruction, overfishing, destructive fishing, unsustainable coastal development, nutrient and sediment loading, sea level rise, frequency and intensity of tropical storms, ocean circulation patterns, and sometimes disease, all have a role to play in coral’s status and survive. As an example, it is established that acidification of the ocean has reduced calcification rates in reef-building and reef-associated organisms by altering seawater chemistry.

For high mountain regions like the Alps, warming expressed by the near surface air temperature over recent decades outpaced the global warming rate, causing continued melting of the glaciers and permafrost (IPCC 2018). Increases in permafrost temperature up to 0.3°C per decade were recorded at depths of about 20 m over the last one to three decades. Warming and thawing of permafrost are expected to increase the risk of rock falls, debris flows and ground subsidence. Thawing of permafrost also affects biodiversity and can contribute to climate change through the release of the greenhouse gases carbon dioxide and methane.

Arctic snow-cover has also continued to change. Most notably, the snow-cover duration has been decreasing rapidly (3–5 days per decade) during the period 1982–2011 (Barichivich et al. 2013). Other changes such as increased potential for unseasonal thaws, early snowmelt, and rain-on-snow events have been revealed (Liston and Hiemstra 2011). These changes have been linked to snow properties and runoff (Semmens et al. 2013), as well as ecosystems and societies (Hansen et al. 2014). Arctic snow-cover has also been studied in several disciplines and put into different disciplinary perspectives (Bokhorst et al. 2016).

Partly due to the highly reflective snow surface, the Arctic has a high risk from UV-B damage for human, animals and vegetations. A long-term declining trend of the stratospheric ozone over the Arctic has been observed. Furthermore, more frequent episodes of extremely low total ozone, particularly during the springtime when new leaves are particularly vulnerable to damage, have been observed.

## Future outlooks and research fronts

Even if carbon emissions were to stabilize today, the atmospheric carbon dioxide concentration increase and global warming will continue, causing inevitable change in the chemistry of the world’s oceans, and continued global warming.

The IPCC (2019) projected a decline of coral reefs by a further 70–90% at 1.5 °C global warming with larger losses (> 99%) at 2 °C. Further changes in non-climatic conditions, such as reduced oxygen levels, will create complex and difficult conditions for coral reefs. Thus, understanding whether and how corals will adjust to keep pace with environmental change has become a research priority.

Looking ahead, glaciers worldwide will be lost and regions with small glaciers, such as the European Alps, can lose most or all glaciers by 2100 (Zekollari et al. 2019). This is of importance to sea level which will rise when ocean water warms and expands in volume. When the melted water of the cryosphere will eventually end up in the oceans, the sea level will rise further.

The Arctic has been and will be a hot spot for climate change, partly due to the so-called Arctic amplification (IPCC 2019). Arctic snowfall is projected to decrease as rainfall makes up more of the precipitation (Bintanja and Andry 2017). Elevated UV-B levels due to ozone depletion are also expected to continue. Current estimates indicate that stratospheric ozone levels will continue to deplete for the next two decades, although there is some uncertainty associated with this projection. New studies looking into the role of UV-B radiation in enhancing the toxicity of certain chemical compounds, particularly those associated with oil spills or petroleum contamination deserve to be further developed.

## Responses from international scientific communities and policy bodies

Since 1980 the international scientific community has worked hard to coordinate research on global environment changes through four global change programmes (Reid et al. 2010). Started in 2009, the International Council for Science (ICSU) led a visioning process for Earth System Research focusing on solutions to environmental problems, leading to the identification of five Grand Challenges for research (Reid et al. 2010). The process inspired the climate researchers under the World Climate Research Programme (WCRP) to develop an initiative for future climate research (Shaprio et al. 2010) and seven priority areas called the WCRP Grand Challenges (Beniston 2013). One of these is “Melting Ice and Global Consequences” which focuses on the cryosphere, a defining feature of high maintains and polar regions.

The Intergovernmental Panel on Climate Change (IPCC) was created in 1988 to bridge the gap between the international scientific communities and police bodies consisting of national governments under the United Nations (UN), through its five assessment reports. In 1992, the UN Conference on Environment and Development held in Rio de Janeiro identified climate change as one of the major environmental challenges facing humankind and the Framework Convention on Climate Change was ratified. Meanwhile, two other major UN conventions, namely the biodiversity and the desertification conventions were also drafted. In 1996 the Kyoto Protocol was established with the aim of reducing anthropogenic carbon emissions.

Recognizing the impact of climate change on humans and ecosystems, world leaders gathered in Paris in 2015 to agree on goals to limit global warming to 2 °C or even 1.5 °C above pre-industrial levels. Unfortunately, this ambition has been challenged by several political setbacks. In 2019, a UN report (WMO 2020) reveals that the world’s climate warming is accelerating, and countries still aren’t fulfilling commitments they made at the UN Paris climate conference in 2015. If we don’t take a collective and quick action, the world would be on course for a 4–5 °C temperature increase by the end of this century. Human induced climate change has been, and remains to be one of the most critical threats to sensitive ecosystems and human society. At the same time, the need to act has never been so clear and urgent.

Concerning the coral bleaching issue, the UN Environment Programme has been running a Regional Seas Programme since 1974. The overarching goal of most regional seas conventions and action plans is to support contracting parties in the protection, conservation and sustainable development of the marine and coastal environment. These are useful, but not sufficient. In the face of global challenges, a global coordination and cooperation is badly needed.

Due to the critical role of the ocean and the cryosphere for life on Earth, the IPCC published a special report on the ocean and cryosphere in a changing climate (IPCC 2019). This report assessed the most recent knowledge about climate changes and climate-related risks of today and in the future in oceans, high mountain areas and polar regions.

The determination that the world’s mountain regions are particularly vulnerable to the unprecedented speed and magnitude of climate change, promoted scientific research, as well as development and implementation of practical solutions for monitoring and adaptation. The scientific research on the climate and environment in the Alps has a long tradition, providing inspirations for other mountain regions such as the Andes and the so-called Third Pole which is the Tibetan plateaus and its surrounding mountain regions (Yao et al. 2019). International programs such as the Third Pole Environment (TPE) have the potential to support and promote regional cooperation across national boundaries.

The Arctic Council, formally established in 1996, is the leading intergovernmental forum promoting cooperation, coordination and interaction among the Arctic States, Arctic indigenous communities and other Arctic inhabitants on common Arctic issues, including climate change and its impacts. Through its working group and the scientific assessments, the Arctic Council has served as a knowledge broker between scientific community and policy world. One successful example is its Arctic Climate Impact Assessment (ACIA) in 2004.

Evidence-based policy making is the success factor for improved environmental conditions. This has been demonstrated by the policy response to deal with the ozone hole. The Nobel prize winning discovery of the causes and mechanisms of the stratospheric ozone reduction made a convincing case for the world’s governments to sign the Montreal Protocol. The implementation of the agreement led to a significant reduction of the atmospheric concentrations of these chemicals and the recovery of the ozone layer over the last 20 years. However, due to the long lifetime of these chemicals, we cannot expect a full recovery of the ozone layer until some 30 years later.

In conclusion, we have the scientific understanding and evidence, as well as some policy bodies and instruments to handle the issues discussed. What is badly needed is to take collective and effective actions. We can make a difference with the choice we are making.
